# Transient Study of Femtosecond Laser–Induced Ge_2_Sb_2_Te_5_ Phase Change Film Morphology

**DOI:** 10.3390/mi12060616

**Published:** 2021-05-27

**Authors:** Wenju Zhou, Zifeng Zhang, Qingwei Zhang, Dongfeng Qi, Tianxiang Xu, Shixun Dai, Xiang Shen

**Affiliations:** 1 Laboratory of Infrared Materials and Devices, Ningbo University, Ningbo 315211, China; zhouwenju000@126.com (W.Z.); m19857840780@163.com (Q.Z.); daishixun@nbu.edu.cn (S.D.); shenxiang@nbu.edu.cn (X.S.); 2 Center for Advanced Laser Manufacturing (CALM), Shandong University of Technology, Zibo 255000, China; 3 College of Mechanical and Electronic Engineering, Chaohu University, Hefei 230000, China; zhangzfxmu@hotmail.com

**Keywords:** laser-induced crystallization, pump-probing technology, pulse laser irradiation

## Abstract

Femtosecond laser-induced crystallization and ablation of Ge_2_Sb_2_Te_5_ (GST) phase change film is investigated by reflectivity pump-probing technology. Below the ablation threshold, the face-centered cubic structure (FCC) state in the central area can be formed, and cylindrical rims are formed in the peripheral dewetting zone due to the solidification of transported matter. The time of surface temperature dropping to the crystallization point needs about 30 ps for 5.86 mJ/cm^2^ and 82 ps for 7.04 mJ/cm^2^, respectively. At higher laser fluence, crystallization GST island structures appear in the central ablation region due to the extremely short heating time (100 ps). Furthermore, crystallization rate is faster than the ablation rate of the GST film, which is caused by different reflectivity.

## 1. Introduction

The quest for non-volatile random-access memories for information storage has led to the exploration of various solutions based on different physical effects. Phase-change memory (PCM) is considered one of the most mature technologies among emerging non-volatile memories. In phase-change memories, each bit of information is stored in a small portion of a phase-change material, which can exist in two different solid states: crystalline and amorphous. The considerable difference in the resistance values of the amorphous (high-resistance) and crystalline (low-resistance) states, which correspond to the logical ‘0’ and ‘1’ states, respectively. Ge–Sb–Te alloys are widely used for data recording based on a fast and reversible amorphous-to-crystalline phase transition [1], which can be achieved by local heating/cooling (either with laser or electrical pulses) at high rates. The phase transformation is accompanied by changes in electrical resistivity and optical reflectively contrast between the two solids, which is used to read data [2,3]. At present, the method of inducing phase change is mainly to realize data reading and erasing by laser thermal or electro-thermal phase transition. Short pulse laser induced phase change in the thin film can improve the speed of information storage [4,5,6]. Moreover, a femtosecond laser pulse can induce such an ultrafast phase change due to the fast displacement of energy on the phase film [7,8]. Since it is intercalation with the material in a short time, it can effectively suppress the thermal and flow mechanical effects of the ablation process [7]. Although the femtosecond laser with the characteristics of “cold processing” and high peak power, the electron temperature can reach thermal equilibrium to tens of thousands of degrees within a few hundred femtoseconds, and completing the electron-lattice relaxation within tens to hundreds of picoseconds. The high temperature can make the material vaporize instantaneously, but the residual heat could still be transferred inside the material, causing phenomena such as crystallization and melting when enough energy is deposited in the material [9,10,11,12]. In this condition, such an ultrafast non-thermal structural change in a phase material offers us the opportunity to develop innovative storage and switching devices.

Fundamentally understanding of laser-induced phase transition and ablation processes is significant in order to better study the properties of phase change film material [13]. The deep understanding of the basic physics mechanism involved in the femtosecond laser ablation of metals is of great importance in terms of improving the efficiency and quality of laser micro-machining and optimizing the processing technology. Understanding the microscopic mechanisms employed in crystallization is crucial to optimize the performance of phase change memory data storage. Several optical detection techniques have been developed to study the interaction of laser materials [14,15]. For example, the pump-probing and time-resolved shadowgraphy has been used to directly observe the ablation process in the picoseconds time regime [16,17], and the reflectivity changes and shock wave emission at certain delay times are respectively captured [18]. However, even in the most common case of phase change thin films, the induced ablation processes and phase change have not been fully studied.

In this work, we investigated the evolution of the surface morphology of the phase change film material induced by the femtosecond laser. With the model combines one-dimensional two-temperature model with classical molecular dynamics method, we can gain the information of the mechanism of laser-material interaction. We selected Ge_2_Sb_2_Te_5_ (GST) as the sample of interest because of its well-known characteristics and have been used as infrared material in some optical devices. We have performed in-situ visualization of femtosecond laser-induced crystallization and ablation processes in the GST film [19,20]. The pump-probing device can help clarify the process of phase transition, and the surface tension induced rim structure, and their dependence on the applied laser fluences [20,21,22]. By studying the relationship between the surface morphologies and the laser fluences, we can make special structures on the surface of GST film. Compared with the traditional annealing and electric pulse methods, the femtosecond laser can greatly shorten the processing period and improve the processing efficiency.

## 2. Materials and Methods

We used the 220 nm thick Ge_2_Sb_2_Te_5_ (GST) film as the sample for the study, it has been fabricated on a SiO_2_ substrate by magnetron sputtering system, using a GST alloy target and a mixture of argon and nitrogen as the sputtering gas. The working pressures and background were prior to 0.3 Pa and 4.8 × 10^−4^ Pa, respectively. The radio frequency (RF) power on the GST target was regular to be 60 W, and the deposition time is 15 min.

**Figure 1:**
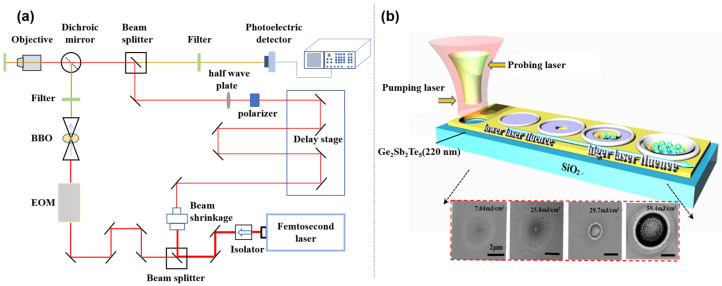
(**a**) The pump-probe technique schematic diagram of femtosecond laser-induced in GST; (**b**) Schematic diagram and SEM pictures of laser processing effect.

The reflectivity pump-probing device showed the evolution of different morphologies during the crystallization and ablation processes after femtosecond laser irradiation. The schematic diagram of the laser-induced GST film crystallization and ablation morphologies is shown in Figure 1. The pump-probing system adopts the Ti: sapphire tunable femtosecond laser Coherent–OPA system (center wavelength: 800 nm, pulse width: 150 fs, frequency: 1000 Hz) of Coherent Company. After the femtosecond laser passes through the beam splitter and the BBO crystal, a 400 nm femtosecond pulse laser is generated as the probing source, and the interval time (time accuracy is ps) between the pumping beam and the probing beam is controlled by a high-precision electric displacement delay stage (step size: 50 nm). The reflection changes of the surface morphology during laser irradiation can be captured at different delay time. The pump beam and probing beam are coaxial and focused by an achromat lens. Both of two beams would been focused to the center of the illuminated area with normal incidence. The probe beam and laser processing beam were measured by the knife-edge µm and 32 µm, respectively. The combination of half-wave plate and a polarizer can continuously adjust the energy of the pump beam. Inserting a dichroic mirror in front of the CCD window to filter out the reflective pump beam. The photodetector obtains the change of the probing beam signal with different delay time, and the signal passes through the lock-phase amplifier and enters the computer for data analysis. The pump-probe technology is used to measure key parameters such as material reflectivity and electron-lattice heating time during the ablation process. To ensure authenticity, at least four signals have been studied

The morphologies of the formed surface structures were characterized by a SEM system (LEO 1530) at an operating voltage of 20 kV. Using the SPI4000/SPA–400 system running in the tapping mode, the micro-nano structure holes were analyzed by atomic force microscope (AFM). Raman spectra were measured using 785 nm CW laser as excitation source which frequency resolution is ±0.1 cm^−1^. The instrument is equipped with a 50× magnifying objective (NA = 0.75), so that we can easily find the processing area.

## 3. Results and Discussion

**Figure 2:**
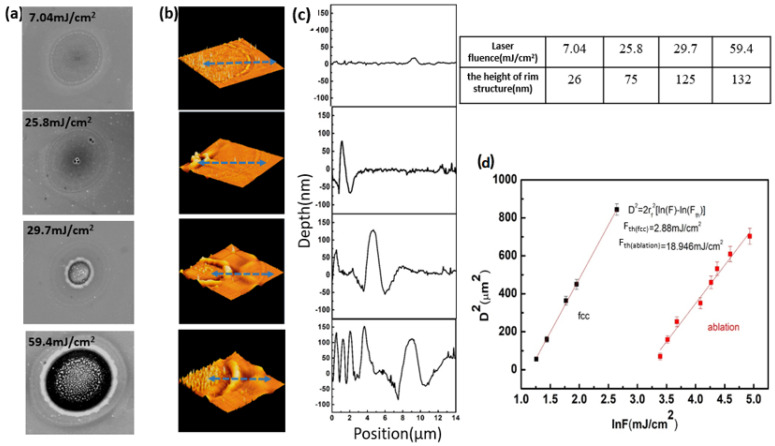
(**a**) Scanning electron microscopy; (**b**) AFM images; (**c**) the cross-sections AFM images; (**d**) Scheme 2. with a different degrees of phase change versus natural logarithm of the pulse fluence ln(F).

During laser processing of GST thin film, two significant states are observed, i.e., crystalline and ablation. Figure 2a gives the normalized correlation of laser fluence to induced region of interest including crystalline and ablation. Due to the laser energy having a Gaussian profile, the morphologies change first at the center and then expand to the surroundings. With laser fluence slightly above the crystalline threshold (2.88 mJ/cm^2^) and below the ablation threshold (18.95 mJ/cm^2^), crystallization line appears in the spot center. Correspondingly, a dark contrast of the center region is visible in SEM image (Figure 2a). The laser irradiation served as an annealing process improved the film quality. When the laser fluence reached the ablation threshold (18.95 mJ/cm^2^), the ablation holes emerge (Figure 2a). The formation of holes is the effect of surface energy minimization once the GST film experienced partial melting. The location is determined by the heterogeneous nucleation. The detailed crossing-sectional properties of the special structures were measured by AFM images as shown in Figure 2b,c and density of the island structures inside the ablation hole also increases with higher fluence (29.7 mJ/cm^2^ and 59.4 mJ/cm^2^). At the center of the network, some dewetted islands formed, which is induced by the complete melting induced localized dewetting. Additionally, owing to the extremely short term of the heating time, it is characterized by the eruption of the melt solidified at an extremely high cooling rate, thereby forming an island structure. For the samples, when the laser fluence is higher than the melting threshold, the SEM images all show a prominent ring structure in the amorphous region. Its appearance indicates that the ring is formed by the destruction of the covering natural oxide layer. Since the melting temperature of GST is lower than that of amorphous silica ($T_{m\left(\alpha -GST\right)}$ = 888 K < $T_{m\left(\alpha -SiO_{2}\right)}$ = 1973 K [23]), this could arise from a local mechanical destruction due to melt flows or due to the volume contraction and expansion of the underlying silicon during melting and subsequent resolidification (mass densities of the solid and the liquid: ρ(s−Si) = 2.32 g/cm^3^ and ρ(1−Si) = 2.52 g/cm^3^ [24]).

Next, we calculated the crystallization and ablation threshold fluences of the GST phase change film though linear curve fitting, as shown in Figure 2d. Above the crystallization and ablation threshold fluence, $F_{th}$, the relationship between the crystallized and ablated spot radius, $r_{a}$, and the laser fluence, F, is described by a Gaussian beam profile, $r_{a}^{2}=2r_{f}^{2}[ln(F)-ln(F_{th})]$, where the 1/e beam radius [25], $r_{f}$, is about 16 µm. If the relationship between $r_{a}^{2}$ and ln(F) is drawn, extrapolation to $r_{a}^{2}$ = 0 will produce crystallization and ablation thresholds. For the different phase transition of GST film, the crystallization and ablation thresholds were calculated as 2.88 mJ/cm^2^, 18.946 mJ/cm^2^, respectively.

**Figure 3:**
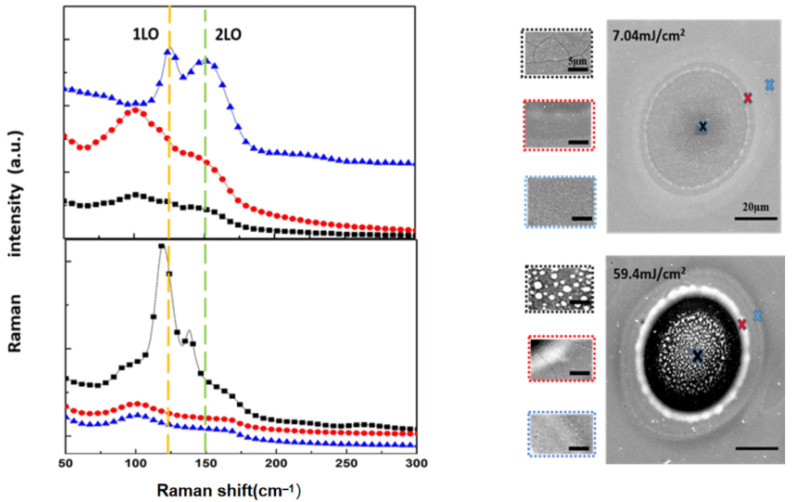
Raman spectra for edge (blue), rim (red), and center (black) regions of dot structures with corresponding SEM images shown on the right, for laser power fluences of 7.04 mJ/cm^2^ and 59.4 mJ/cm^2^, respectively.

**Figure 4:**
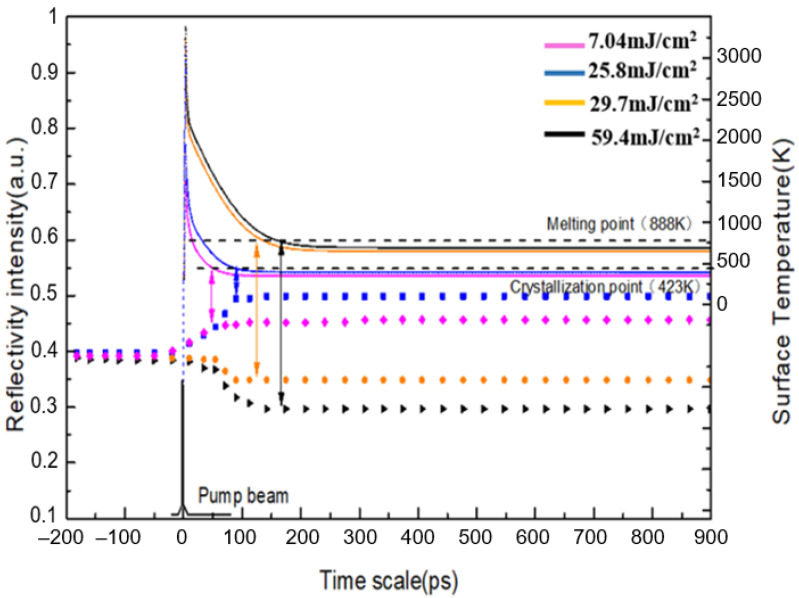
The pump-probing reflectivity signals of femtosecond laser induced crystallization and ablation of Ge_2_Sb_2_Te_5_ with difference laser fluences in scatter-points, and the corresponding simulated surface temperature as a function of time for the applied laser fluences in solid lines.

We use the Raman spectra to better show the phase transition of GST under different laser fluences. Figure 3 shows the Raman spectra measured at different processing area positions (center, rim and edge) at different laser fluences, respectively. The spectrum with a wide characteristic peak at 150 cm^−1^ and a lower peak in the 120 cm^−1^ is the typical peak of amorphous GST film [26]. After laser irradiation (7.04 mJ/cm^2^), at the rim and the center areas, the bimodal position shifts to 158 cm^−1^ and 108 cm^−1^, respectively corresponding to the oscillation of the Ge–Te polar bond and the covalent bond of the atom in the tetrahedron structure, resulting in the formation of the face-centered cubic structure (FCC) crystallization state [27]. When the laser fluence is 59.4 mJ/cm^2^, by analyzing the peak position, the edge and the rim of the spot can be clearly seen as the FCC crystallization state. In the center area, the laser-induced island structures become crystallization state [28]. There were both three phenomena in one light spot. When the laser energy is 7.04 mJ/cm^2^ (low power), there are some crystalline particles and micro-cracks in the center of the spot (black fork). Due to the Gaussian distribution of laser energy, the energy in the center of the spot is the highest. The film in the center of the spot starts to melt firstly. Driven by the force of the light field, heat transfer and surface tension, the molten part forms a ring-shaped protrusion on the edge of the spot (red fork). The energy of the rim of the spot is the weakest, which can only cause crystallization and produce some fine grains (blue fork). When the laser energy is 59.4 mJ/cm^2^ (high power), more energy is deposited on the GST surface. It can be seen from Figure 4 that when the energy decreases to the crystallization temperature, the crystallization rate corresponding to high energy is far less than that corresponding to low energy. Slower cooling rate leads to lower degree of under cooling. While the degree of under cooling of low power is higher. In the case of a high degree of under cooling, the nucleation rate increases, which is faster than the nucleation growth rate. Affected by the heat deposition and transfer, the degree of under cooling of the edge of the spot will also be higher than the center of the spot. Therefore, small grains are obtained at the edge of the light spot. While in the case of low degree of under cooling, the nucleation rate slows down and the nuclei tend to grow, which leads to the formation of large crystalline particles in the center of the light spot (black fork). Due to high peak power of the laser, the material in the center of the spot is vaporized instantly, and the island crystalline particles in the center are caused by the transfer of residual heat. The principle of morphology of red and blue fork of high power is the same of lower power.

The reflection probing brings to light the transient dynamics of laser interaction with GST. Figure 4 shows the time-resolved regular reflection for different laser fluences. In the laser-induced crystallization state, the rise in low fluence can be inferred from the order of the atomic arrangement, so that the film changes from the amorphous state to the crystalline state, resulting in an increase in reflectivity [29]. When the fluence of the pump laser is higher, the reflection drops directly to a lower state. The probing signal drops at higher pump fluence can be believed as the effect of the ablation processing, which indicates a weaker reflection. Above the crystallization threshold the area of the crystallized region increases continuously, leading to a smooth increase in reflection due to growth in the size of the crystalline region. Above the ablation threshold, the center of the laser spot melts the layer, and the subsequent quenching leads to a small amorphous mark (some dewetted islands) in the middle. The drop in reflectivity is caused by hole ablation. The reflection therefore decreases as the power is further increased. Therefore, the measurement of reflection intensity in turn proves the dynamics of the ablation processing. In addition, the variation trend of surface temperature after laser irradiation is also studied, as shown in Figure 4. During pulse laser heating, assuming that the lattice and the electron systems can be characterized by a lattice temperature, $T_{l}$, and an electron temperature, $T_{e}$, respectively. Therefore, the energy conversion follows the one-dimensional dual-temperature model proposed Qiu and Tien [16]. The width of femtosecond laser pulse is much smaller than the relaxation time between electron and phonon, and the laser ablation is regarded as non-equilibrium ablation. The process of radiating material by picosecond laser pulse is a complex ablation mechanism, including non-equilibrium ablation and equilibrium ablation. The width of the nanosecond pulse is much larger than the relaxation time of electrons and phonons, it can be regarded as a thermal equilibrium ablation process. According to the characteristics of the two ablation states, non-equilibrium and equilibrium, the two-temperature theoretical model of pulse width from femtosecond to nanosecond can be summarized as:

(1) $$C_{e}\frac{\partial }{\partial t}T_{e}=k_{e}\exp\left[-a\frac{\tau_{R}}{\tau_{L}}\right]\frac{\partial^{2}}{\partial x^{2}}T_{e}-g\left(T_{e}-T_{l}\right)+\left(1-R\right)\alpha_{b}I_{0}\left(t\right)\exp\left(-\alpha_{b}x\right)$$

(2) $$C_{l}\frac{\partial }{\partial t}T_{l}=g\left(T_{e}-T_{l}\right)$$

Equations (1) and (2) show the heat transfer process of electron and lattice respectively. The formula $\left(1-R\right)\alpha_{b}I_{0}\left(t\right)\exp\left(-\alpha_{b}x\right)$ is expressed as the heat source of the laser, the coupling term between electrons and phonons is $g\left(T_{e}-T_{l}\right)$*. T_l_* is the temperature of the crystal lattice, $T_{e}$ is the temperature of electrons, $C_{e}$ is the specific heat capacity of electrons, $C_{l}$ is the specific heat capacity of the crystal lattice, g is the coupling coefficient between electrons and phonons, $k_{e}$ is the thermal conduction of electrons, and R is the surface reflection of the material. $\alpha_{b}$ is the linear absorption coefficient of the material, a represents a parameter determined by different materials, the value of which is different for different materials. It is usually determined by a combination of experiment and theory. The physical properties of materials are affected by electronic excitation. When the electron temperature is less than 3000 K, the influence of electron excitation can be ignored.

The ratio of the width of the laser pulse to the relaxation time of the electron phonon is an important rule. When the pulse width is much smaller than the electron phonon relaxation time, it is a non-equilibrium ablation state, the two-temperature theoretical model can be simply regarded as a general two-temperature function equation. When the pulse width is much larger than the electron phonon relaxation time, the ablation process can be regarded as an equilibrium ablation process, and the two-temperature theoretical model can be regarded as a one-dimensional heat transfer function. When the pulse width is similar to the electron phonon relaxation time, the ablation process includes both non-equilibrium ablation and equilibrium ablation, and this process is more complicated.

When the pulse width is much smaller than the electron phonon relaxation time: $\exp\left[-a\frac{\tau_{R}}{\tau_{L}}\right]\to 0$, Equations (1) and (2) can be transformed as follows:

(3) $$C_{e}\frac{\partial }{\partial t}T_{e}=-g\left(T_{e}-T_{l}\right)+\left(1-R\right)\alpha_{b}I_{0}\left(t\right)\exp\left(-\alpha_{b}x\right)$$

(4) $$C_{l}\frac{\partial }{\partial t}T_{l}=g\left(T_{e}-T_{l}\right)$$

Equations (3) and (4) are classical two-temperature theoretical model. The Gaussian distribution of laser energy can be expressed as:

(5) $$I_{0}\left(t\right)=I_{0}\exp\left[-\frac{\left(4ln2\right)t^{2}}{{\left(\tau_{p}\right)}^{2}}\right]$$

where $I_{0}$ represents the density of the maximum laser pulse energy. The expression of the laser heat source used in the experiment is as follows:

(6) $$S\left(x,t\right)=0.94\frac{1-R}{t_{p}\delta }J\cdot \exp\left[-\frac{x}{\delta }-2.77{\left(\frac{t}{t_{p}}\right)}^{2}\right]$$

where $t_{p}$ is pulse width. The initial conditions of Equations (1) and (2) are as follows:

(7) $$-k_{e}\frac{\partial T_{e}}{\partial t}{|}_{x=0}=\left(1-R\right)I_{0}\left(t\right)$$

(8) $$-k_{e}\frac{\partial T_{e}}{\partial t}{|}_{\delta }=T_{0}$$

δ is the thermal penetration depth of the material. The electron density is small when the pulse energy is small. The depth of energy transfer is $\delta =1/\alpha_{b}$. When the laser pulse with high energy density, the depth of energy transfer is $\delta =\sqrt{\frac{k_{e}}{T_{e}}\tau_{R}}$. The initial boundary conditions for both the electron and lattice system can be defined as $T_{e}\left(x,-2t_{p}\right)=T_{l}\left(x,-2t_{p}\right)=T_{0}$ and $T_{0}$ = 300 K [30]. All simulated materials are GST, and their physical constants are listed in Table 1.

At lower laser fluence, the time of the surface temperature dropping to the crystallization point of the material is about 50–70 ps, which corresponds well with the experiment results. As the laser energy density increases, the lattice temperature reaches the highest first and then gradually decreases. At high fluence, it takes more time for the lattice temperature to drop to the melting point of the material, and the surface reflectivity of the material will slowly decrease (120 ps for 29.7 mJ/cm^2^, 170 ps for 59.4 mJ/cm^2^). The surface temperature was heated up firstly and decreased to its steady-state in 120 ps and 170 ps respectively, causing the melting pool in the center, resulting in the material diffuses and gathers surround the crater. Due to the higher temperature of the surface at the center of the melt pool, and the absorbed energy due to the high temperature gradient, surface tension-driven flow is caused. Thus, because of the diminishing surface tension with increasing temperature for liquid metals, material should be transported radially outward by the positive surface tension gradient at higher laser fluence. Thus, it can be clearly seen that the crystallization rate (the rate is the slope of the intersection of the surface temperature curve and crystallization temperature or melting temperature curve (the dotted line)) is faster than the ablation rate, which results in the formation of the crystal GST island structures in the center area.

## 4. Conclusions

In conclusion, we have carried out in-situ visualization of femtosecond laser-induced crystallization and ablation processes in thin GST film. The pump-probing setup helped elucidate the transient breakup of the melt rim, island structure and ablation pool. Additionally, the trends of the surface temperature after laser irradiation are also investigated.

## Figures and Tables

**Table 1:** Parameters for Ge_2_Sb_2_Te_5_ used in heat calculation [16,31,32].

Ge_2_Sb_2_Te_5_ (Parameters)	Values
Initial temperature (*T_0_*)	300 K
Lattice heat capacity (*C_i_*)	1.29 × 10^6^ J m^−3^ K^−1^
Electron heat capacity (*C_e_*)	210 J Kg^−1^ K^−1^
Electron-phonon coupling factor (*G*)	2.6 × 10^16^ W m^−3^ K^−1^
Amorphous reflection coefficient (*R_1_*)	0.35
Crystallization reflection coefficient (*R_2_*)	0.5
